# Placozoan secretory cell types implicated in feeding, innate immunity and regulation of behavior

**DOI:** 10.1101/2024.09.18.613768

**Published:** 2024-12-04

**Authors:** Tatiana D. Mayorova, Thomas Lund Koch, Bechara Kachar, Jae Hoon Jung, Thomas S. Reese, Carolyn L. Smith

## Abstract

Placozoa are millimeter-sized, flat, irregularly shaped ciliated animals that crawl on surfaces in warm oceans feeding on biofilms, which they digest externally. They stand out from other animals due to their simple body plans. They lack organs, body cavities, muscles and a nervous system and have only seven broadly defined morphological cell types, each with a unique distribution. Analyses of single cell transcriptomes of four species of placozoans revealed greater diversity of secretory cell types than evident from morphological studies, but the locations of many of these new cell types were unknown and it was unclear which morphological cell types they represent. Furthermore, there were contradictions between the conclusions of previous studies and the single cell RNAseq studies. To address these issues, we used mRNA probes for genes encoding secretory products expressed in different metacells in *Trichoplax adhaerens* to localize cells in whole mounts and in dissociated cell cultures, where their morphological features could be visualized and identified. The nature and functions of their secretory granules were further investigated with electron microscopic techniques and by imaging secretion in live animals during feeding episodes. We found that two cell types participate in disintegrating prey, one resembling a lytic cell type in mammals and another combining features of zymogen gland cells and enterocytes. We identified secretory epithelial cells expressing glycoproteins or short peptides implicated in defense. We located seven peptidergic cell types and two types of mucocytes. Our findings reveal mechanisms that placozoans use to feed and protect themselves from pathogens and clues about neuropeptidergic signaling. We compare placozoan secretory cell types with cell types in other animal phyla to gain insight about general evolutionary trends in cell type diversification, as well as pathways leading to the emergence of synapomorphies.

## Full Text Availability

The license terms selected by the author(s) for this preprint version do not permit archiving in PMC. The full text is available from the preprint server.

