# Supplementary figures and images for: Placozoan secretory cell types implicated in feeding, innate immunity and regulation of behavior
Source: bioRxiv. 2025 Jan 16:2024.09.18.613768. Originally published 2024 Sep 22. Preprint. [Version 3] doi: 10.1101/2024.09.18.613768 (PMC11452194; doi:10.1101/2024.09.18.613768)

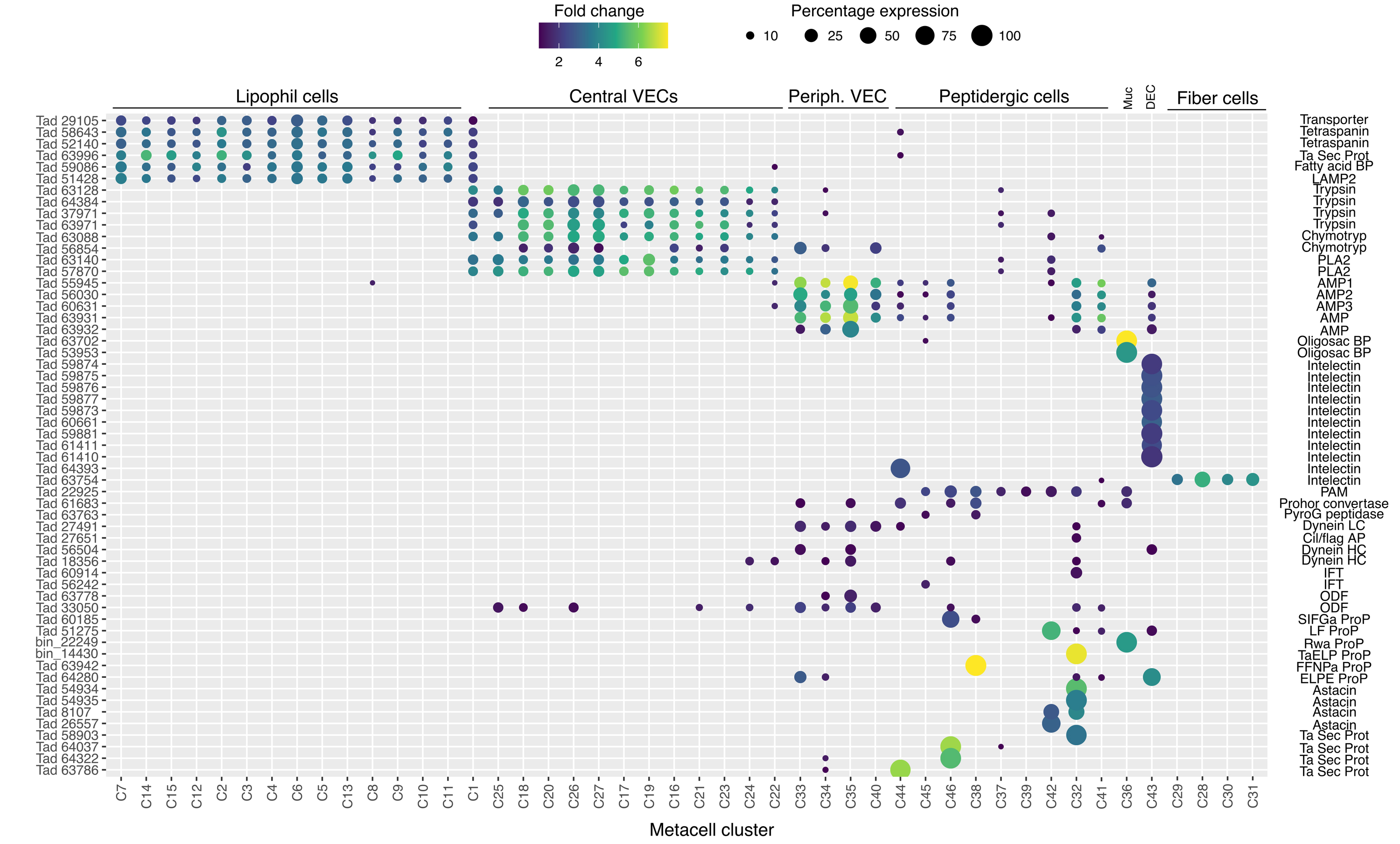

Supplement: Supplement 1 — S1 Fig. Expression of selected genes across T. adhaerens metacells (columns) identified by single cell RNA sequencing. Data are from [30]. Cell types are identified based on data from the present study and [6,18,24,30]. Dot color represents fold change of gene expression and dot size represents percentage of the total UMI in the given metacell. [file media-1.jpg]

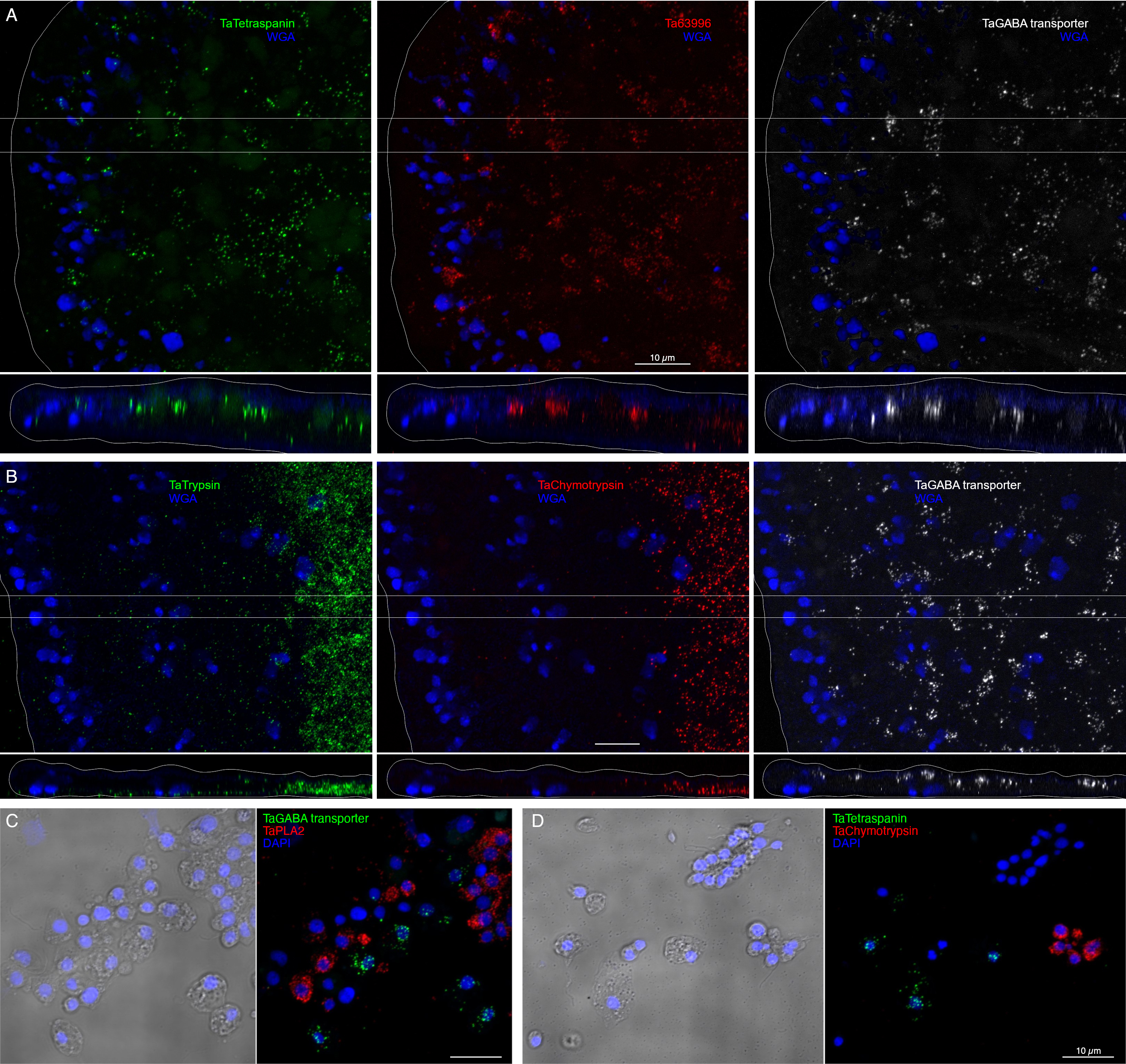

Supplement: Supplement 2 — S2 Fig. Color separated FISH images of the micrographs shown in Fig.2. (A, B) Separated channels corresponding to horizontal (xy) and vertical (xz) projections of color-merged FISH images of TH1 wholemounts in Fig 2A and B. (C, D) Separated DIC and fluorescence channels of the merged images of dissociated cells in Fig 2C and D. [file media-2.jpg]

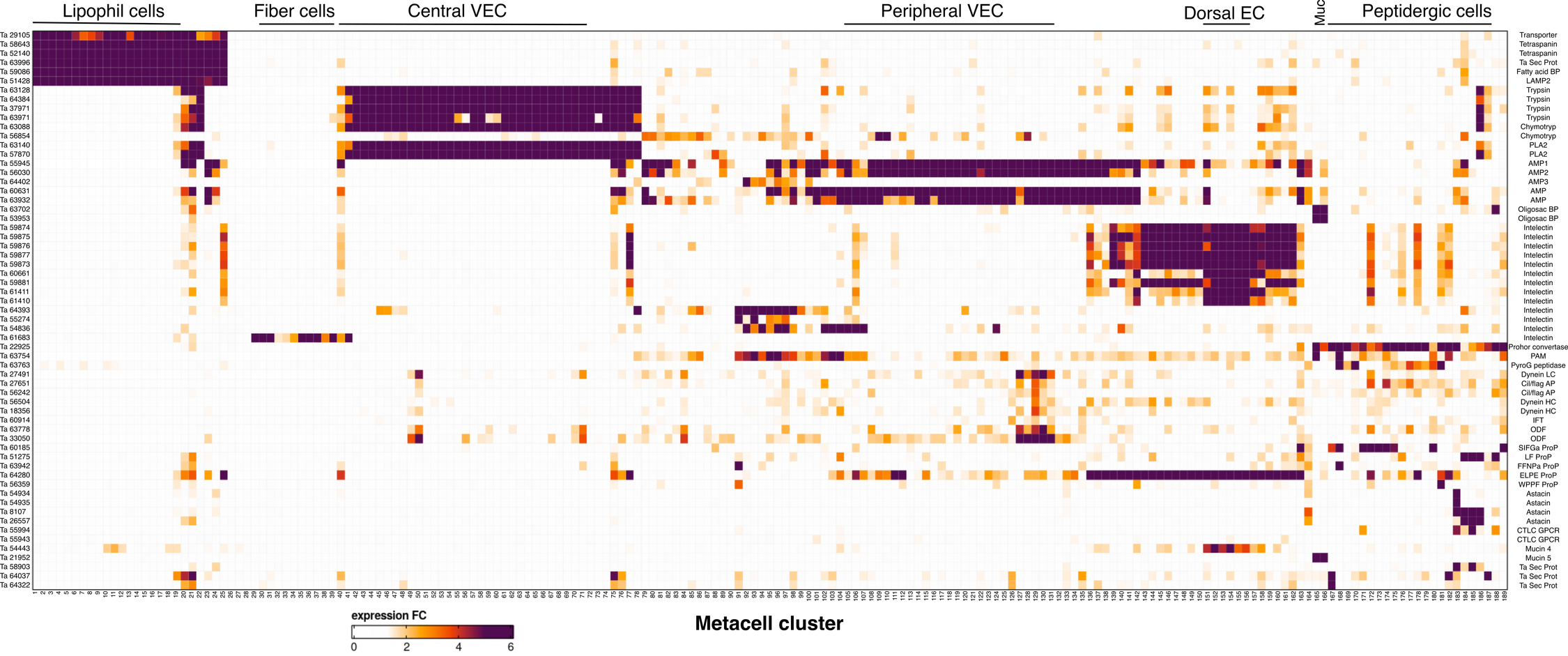

Supplement: Supplement 3 — S3 Fig. Normalized expression of selected genes across metacells for TH1, TH2, HH13, and CH23. Cell types are identified based on data from the present study and [6,18,30]. Expression data from: https://sebelab.crg.eu/placozoa_cell_atlas/ [file media-3.jpg]

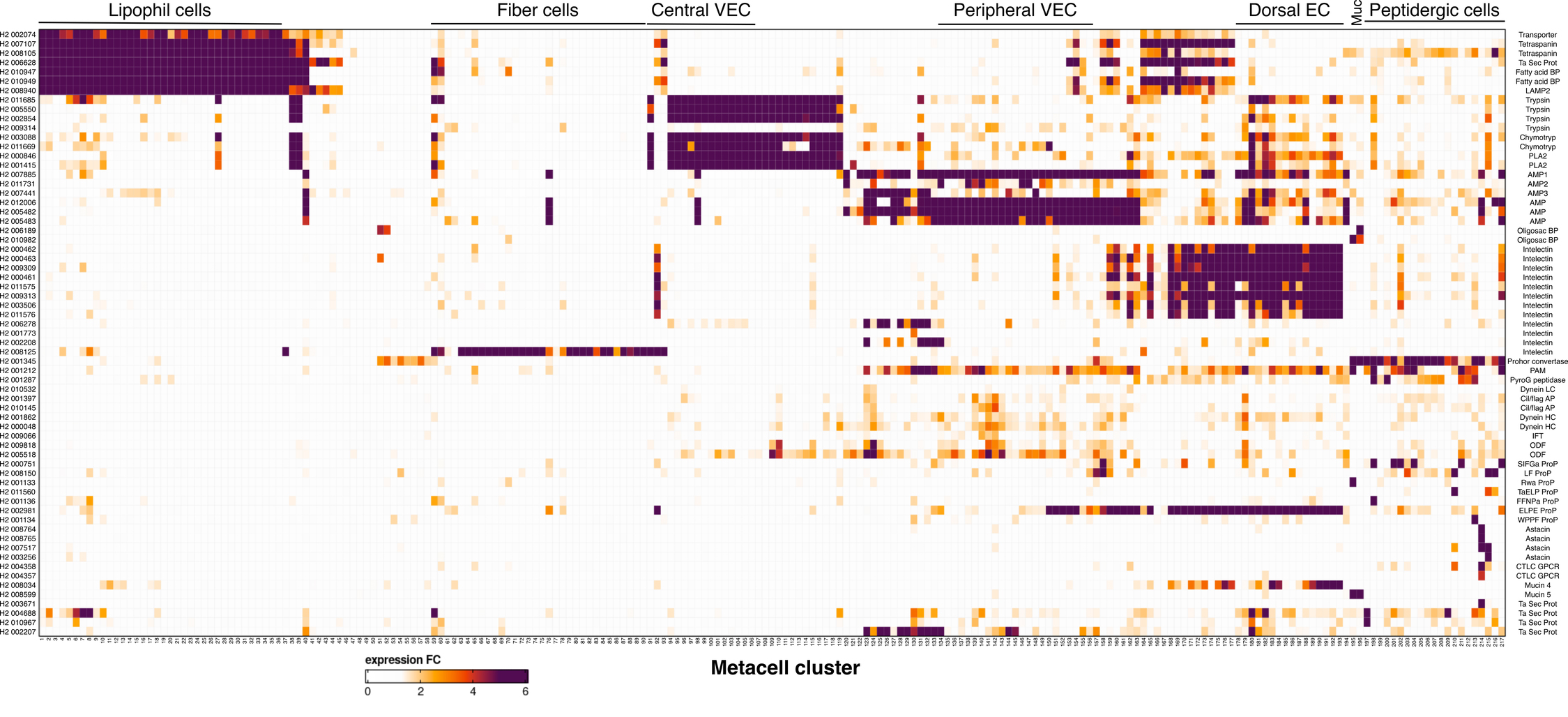

Supplement: Supplement 4 — S4 Fig. Normalized expression of selected genes across metacells for TH1, TH2, HH13, and CH23. Cell types are identified based on data from the present study and [6,18,30]. Expression data from: https://sebelab.crg.eu/placozoa_cell_atlas/ [file media-4.jpg]

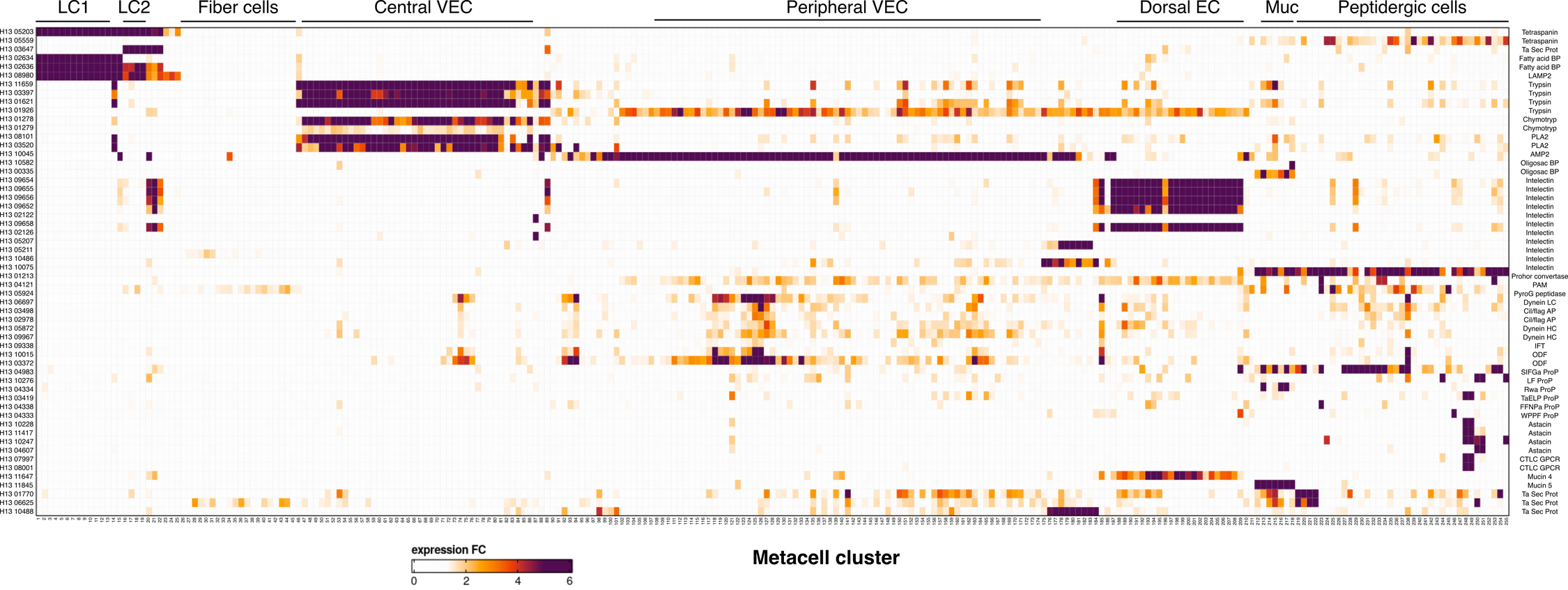

Supplement: Supplement 5 — S5 Fig. Normalized expression of selected genes across metacells for TH1, TH2, HH13, and CH23. Cell types are identified based on data from the present study and [6,18,30]. Expression data from: https://sebelab.crg.eu/placozoa_cell_atlas/ [file media-5.jpg]

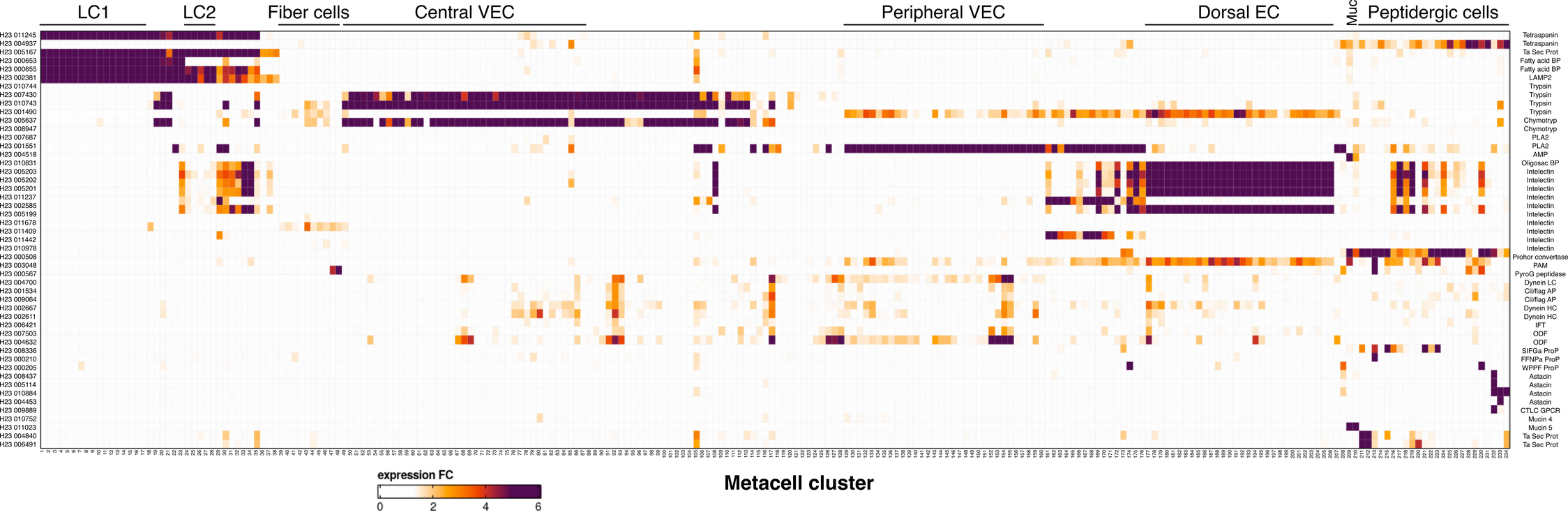

Supplement: Supplement 6 — S6 Fig. Normalized expression of selected genes across metacells for TH1, TH2, HH13, and CH23. Cell types are identified based on data from the present study and [6,18,30]. Expression data from: https://sebelab.crg.eu/placozoa_cell_atlas/ [file media-6.jpg]

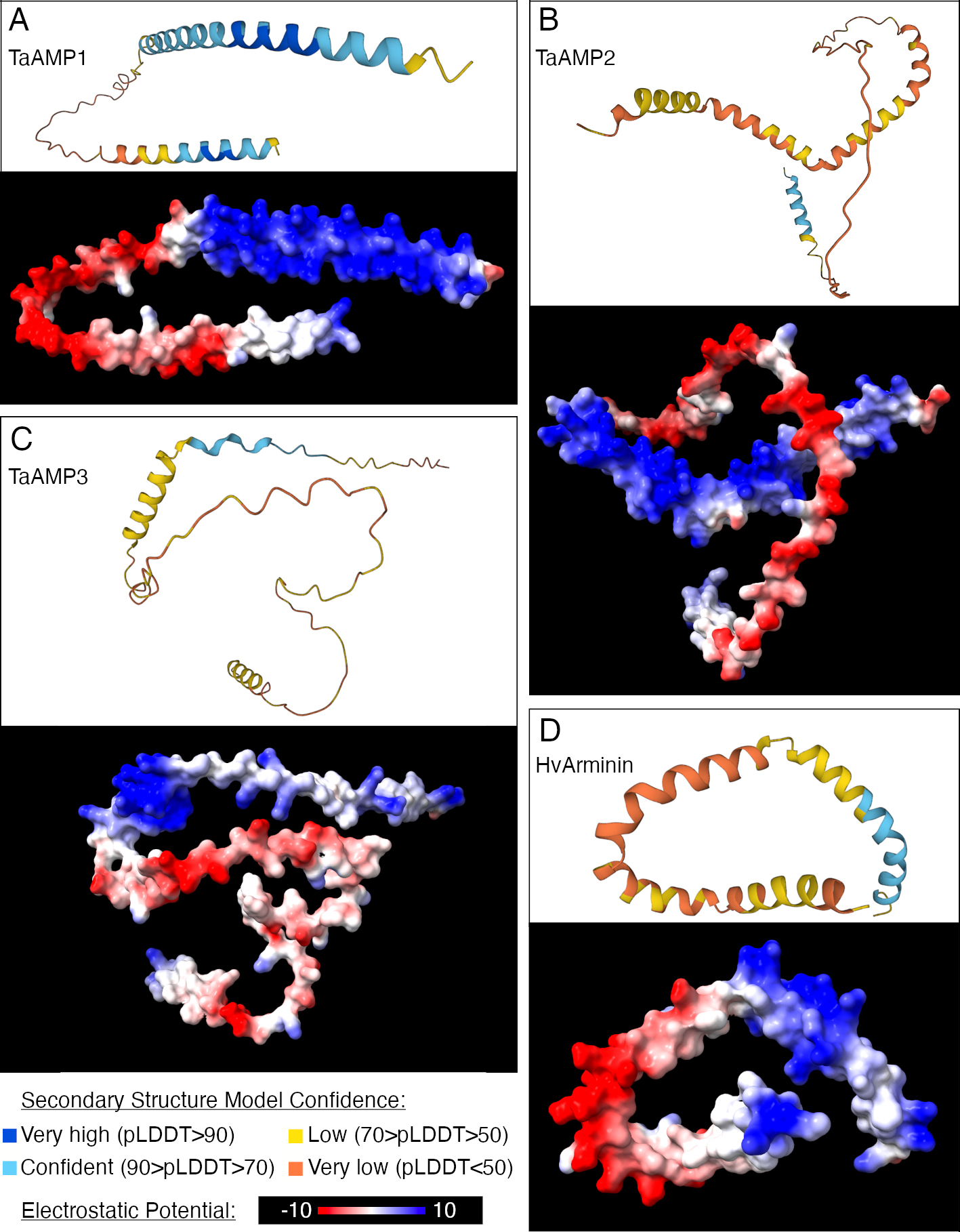

Supplement: Supplement 7 — S7 Fig. Secondary structure predictions and electrostatic potential maps for putative Trichoplax AMPs (AMP1 (A), AMP2 (B), and AMP3 (C) and Hydra vulgaris arminin (D). Secondary structures are predicted with Alpha Fold and peptide surfaces are colored by electrostatic potential with ChimeraX. [file media-7.jpg]

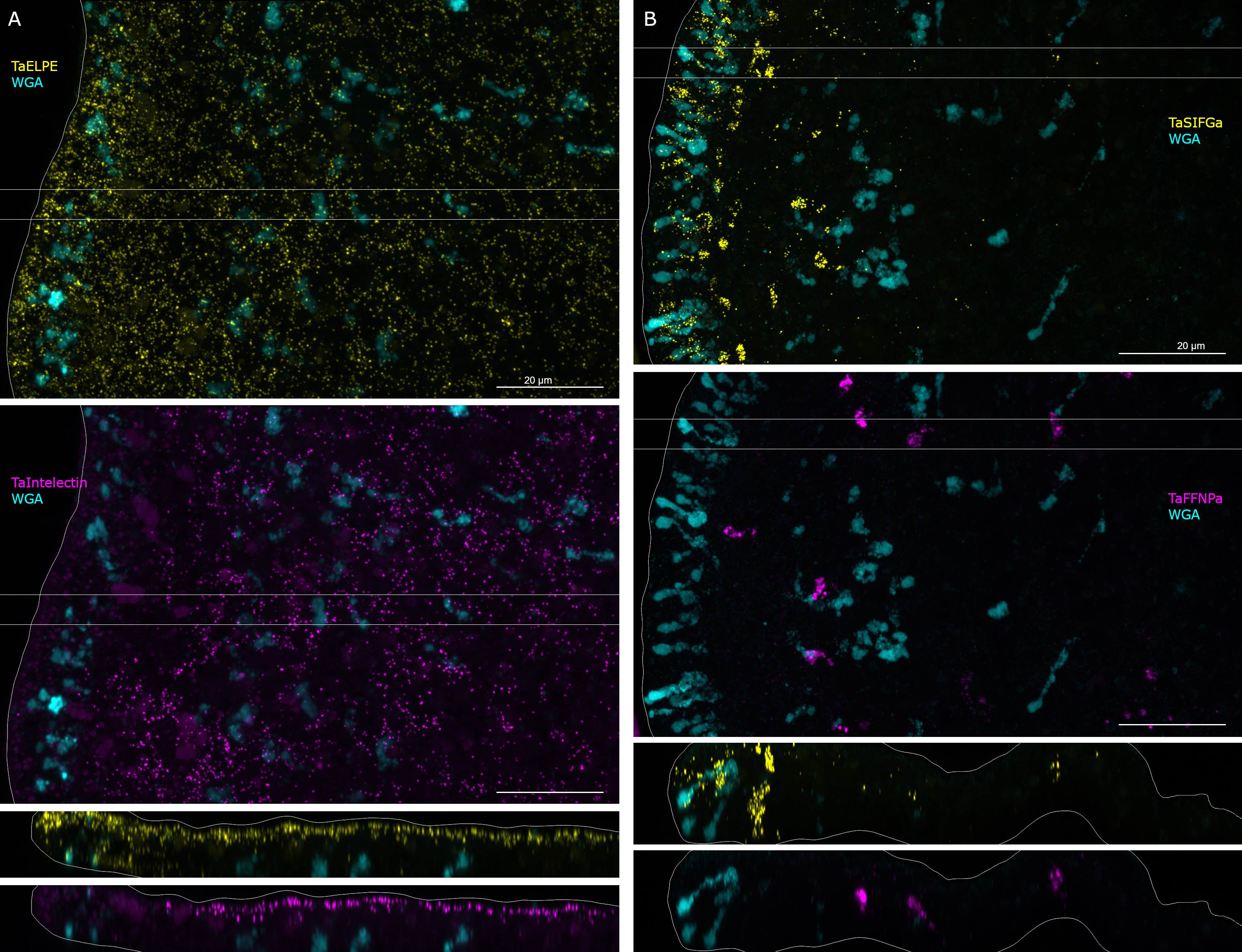

Supplement: Supplement 9 — S9 Fig. Color separated FISH images of TH1 wholemounts corresponding to horizontal (xy) and vertical (xz) of color-merged FISH images in Fig 5F (A) and Fig 6F1 (B). [file media-9.jpg]

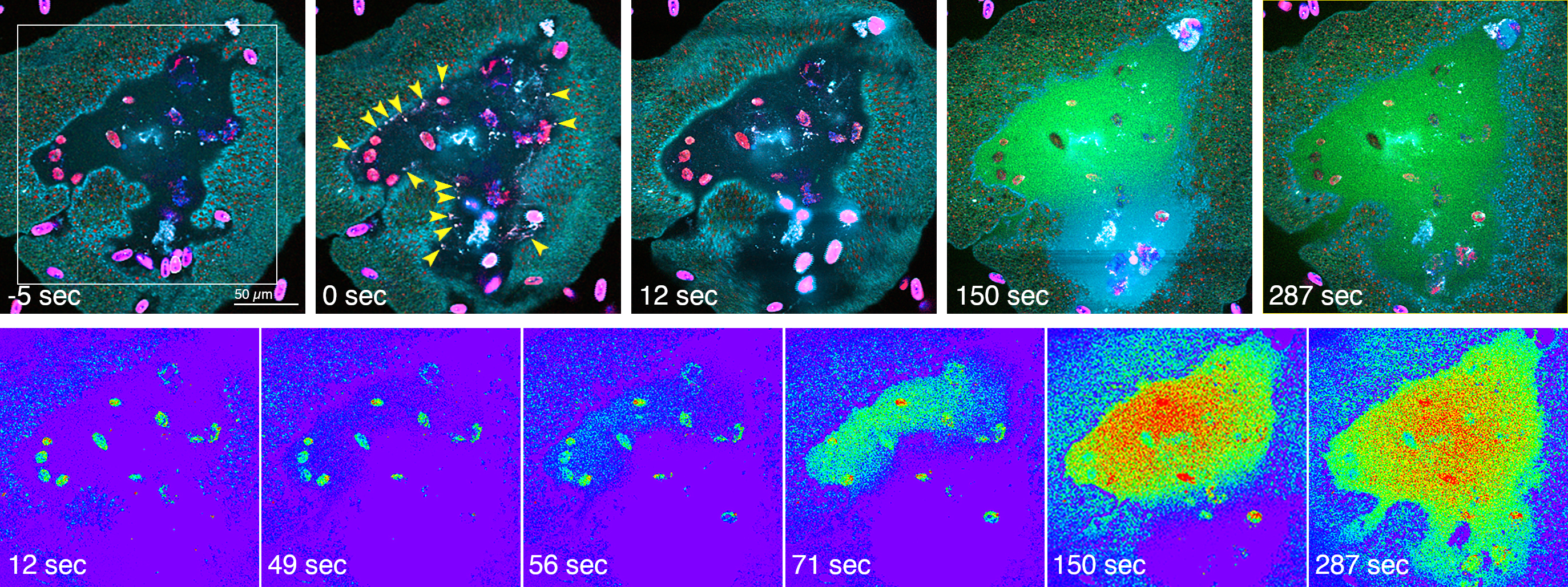

Supplement: Supplement 10 — S10 Fig. Secretory behaviors of Trichoplax feeding on R. salina algae. Lipophil cell granules were labeled with LipidTOX (red). The fluorescent membrane dye FM1–43 (cyan) was added to the seawater to label cell membranes and the contents of LC granules. BZiPAR, a fluorescent indicator of trypsin activity (green), was added to detect secretion of trypsin. Algae are visible by autofluorescence and FM1–43 staining (pink in merged images). At the beginning of the sequence, the peripheral part of the animal was closely attached to the substrate, while the central part was invaginated, forming a feeding pocket enclosing algae. At t=0 sec, lipophil granule secretion was evident due to the appearance of small FM1–43-stained spots (cyan/white; arrowheads) in the feeding pocket. At t=12 sec, several algae (pink) in the feeding pocket were lysed and released material stained by FM1–43 (cyan). By 150 – 287 sec, trypsin activity was evident in the feeding pocket and the lysed algae were decomposing. Bottom panels show intensity-coded images of BZiPAR trypsin activity indicator at sequential timepoints for the boxed region. [file media-10.jpg]
