# Supplementary material for: Placozoan secretory cell types implicated in feeding, innate immunity and regulation of behavior
Source: bioRxiv. 2025 Jan 16:2024.09.18.613768. Originally published 2024 Sep 22. Preprint. [Version 3] doi: 10.1101/2024.09.18.613768 (PMC11452194; doi:10.1101/2024.09.18.613768)
Supplement: Supplement 8 — S8 Text. Placozoan prepropeptides, predicted cleavage sites and processing motifs. Underlined regions represent predicted signal peptides. Highlighted yellow represents the mature peptide region, green represents prohormone convertase cleavage sites, cyan represents pyroglutamate, and magenta represents C-terminal amide. [file media-8.docx]

Peptide region

Amidation

Pyro-glutamination

Cleavage site

>TH1 ELPE prepropeptide

MRSIILICLLFLFAAKVNSESFDSDDKRDLFSDEHQRNNDENVVEDGASISRQFASENDEDNNEDQIPPLGKSFELPEHRRGKSFEFPEHRRGKSFEFPERRRGKSFELPERRRGKSFELPERRRGKSFELPERRRGKSFELPERRRGKSFEFPEHRRGKSFEFPLNVLFQFGNLFRDVLARREGEIKQ

>TH2 ELPE prepropeptide

MRSIILICLLFLFAAKVNSESFDSDDKRDLFSDEHQRNNDENVVEDGASISRQFASENDEDNNEDQIPPLGKSFELPERRRGKSFEFPEHRRGKSFEFPEHRRGKSFELPERRRGKSFELPERRRGKSFELPERRRGKSFELPERRRGKSFEFPEHRRGKSFEFPVRTQTLEIIKLG

>TH1 LF prepropeptide

MRTILVFTLLVVAVSCRAISKNTDEKSKKPKKTEPKLMIGYPLFKKEDLDSQGYALFRKDDSQGYPLFRKDDSQGYPLFRKDDSQDGYALFRKDDSQPGHALFRKDDSQDGYALFRKDAQNGNSILYGHPLFKKEDQDGELSEKADTPLFKKEDSQSADSKKPIIIWKRDGPSSDSEIPMILFKKRQDDDSEKSEAKNVVSWFSQRDTRKQGFIPFKRGHKRLSYIPNSNPFKKIFLGDLSSRSEKMA*

>TH2 LF prepropeptide

MRTILVFTLLVVAVSCRAISKNTDDDTQETTKMETEPKPKLMIGYPLFKKEDLDSQDGYALFRKDDSQPGHALFRKDDSQDGYALFRKDARSENPSNIGHPLFKKEAQNGNSILYGNPLFKKEDQDGELSEKADTPLFKKEDSQSADSKRPIIIWKRDGPSSDSEIPMILFKKRQDDDSEKSEAKNVVSWFSQRDTEKQGFIPFKRGQKRLSYIPNSNPFKKIFLGDLSSRSEKMA*

>HH13 LF-1 prepropeptide

MRTLLIFVLLAIACALVNCRALEDESESWMAKRDYLLARDTKNKKKKTKLNTAAKIGFLLFKKADKLAERDFEDSLFRKSDQNSEETPAVLENIPIFRKSDRNPPDLLLFKKSDQTGGNNLFDPFKRRGIIQHGGYPWNG*

>HH13 LF-2 prepropeptide

MNKINLVTLYIIVAYAIIASSQARNVKWSRSTGTNTHNRESKLPTTFWNGNPSGTGFALFKKADDSKHLNEANRLRTPDGMGYAMFKKVHNNAHFMDKNKLKMPPGVGLPLFKKTQDSKSRKSQVANPPGFNLALFKKAQHDKKLKEDNYQFSDDSGIGLPLFKKAQHDKKPKEDNHQFSDDSGIGLPLFRKAQHDKK

>TH1 SIFGamide prepropeptide

MKQIALIFFLTAAIVFATVNAEGNLESIFNAKREDQANLKSIFGGKREDQANLKSIFGGKREDQANLKSIFGGKREDQANLKSIFGGKREDQANLKSIFGGKREDQANLKSIFGGKREDQANLKSIFGGKREDQANLKSIFGGKREDQANLKSIFGGKREDQANLKSIFGGKREDQANLKSIFGGKREDQANLKSIFGGKREDQANLKSIFGGKRDDQANLKSIFGGKRDDQANLKSIFGGKREDQANLKSIFGGKRDDQANLKSIFGGKREDQANLKSIFGGKREDQANLKSIFGGKREDQANLKSIFGGRREDQANLKSIFGGKREDQANLKSIFGGKREDQANLKSIFGGKREDQANLKSIFGGKREDQANLKSIFGGKREDQANLKSIFGGKREDQANLKSIFGGRREDQANLKSIFGGKREDQANLKSIFGGKREDQANLKSIFGGKREDQANLKSIFGGKREDQANLKSIFGGKREDQANLKSIFGGKREDQANLKSIFGGKREDQANLKSIFGGKREDQANLKSIFGGKRKDRANSKKKFGCKCKGRGNMKSMLGGKREDQANLKSIFDGKREDQANLKSIFGGKREDQANLKSIFGGKRGDQANLKSIYGGKREDQANLKSIYGGKREDQANLKSIFGGK

>TH2 SIFGamide prepropeptide

MKQIALIFFLTAAIVFATVNAEGNLESISNAKREDQANLKSIFGGKREDQANLKSIFGGKREDQANLKSIFGGKREDQANLKSIFGGKREDQANLKSIFGGKREDQANLKSIFGGKREDQANLKSIFGGKREDQANLKSIFGGKREDQANLKSIFGGKREDQANLKSIFGGKREDQANLKSIFGGKRENQANLKSIFGGKREDQANLKSIFGGKRDDQANLKSIFGGKRDDQANLKSIFGGKRDDQANLKSIFGGKREDQANLKSIFGGKREDQANLKSIFGGKREDQANLKSIFGGKREDQANLKSIFGGRREDQANLKSIFGGKREDQANLKSIFGGKREDQANLKSIFGGRREDQANLKSIFGGKREDQANLKSIFGGKREDQANLKSIFGGRREDQANLKSIFGGKREDQANLKSIFGGKRGDQANLKSIFGGKREDQANLKSIFGGKREDQANLKSIFGGKREDQANLKSIFGGMREDRGNVKSMFVGKREDQANLKSIFGGKREDQANLKSIFGGKREDQANLKSIFGGKRGDQANLKSIFGGKRADQANLKSIFGGK

>HH13 TVWGamide prepropeptide

MKSINIIFLTAAILLVSVSAGRRHDDLHKKEDTVWGGRRSDDSQRTGANLQTVWGGRRSDDSQRTGANLQTVWGGRRSDDSQRTGANLQTVWGGRRSDDSQRTGANLQTVWGGRRSDDSQRTGANLQTVWGGRRSDDSQRTGANLQTVWGGRRSDDSQRTGANLQTVWGGRRSDDDSQRTGANLQTVWGGRRSDDSQRTGANLQTVWGGRRSDDDSQRTGANLQTVWGGRRSDDSQRTGANLQTVWGGRRSDDSQRTGANLQTVWGGRRSDDDSQRTGANLQTVWGGRRSDDSQRTGANLQTVWGGRRSDDSQRTGANLQTVWGGRRSDDSKGLVPTYKLYGVDEEAMIHKRQEPTYKLYGVDEEAMIHKRQEPTYKLYGVDEEAMIS

>CH23 SFFGamide prepropeptide

MKSIYIIFFAATIVFASVNADDENVKDYFNERDNDFDVEALYEYDSKRDDDLQKKHGVNLKTFFGGKREDLQRKTGVNLKTFFGGKRDDDLQKKHGVNLKTFFGGKRDDELRKEHSVNLKNFFGREYGDLTPEVQAKTRDLTEALVALVKEIEKFVASRPNLKSFFGGKRDDADDLQRKTGVNLKTFFGGKRDDLQKKTGVNLKTFFGGKRDDLQKKTGVNLKTFFGGKRDDLQKKTGVNLKTFFGGKRDDLQRKTGVNLKTFFGGKRDDLQK

>TH1 endomorphin-like peptide prepropeptide

MDHKIKILALIVIAVAGLSSGKSMDKNGRNSVSLWTSAARDSKLAERNDQRKGYIYWETKRDENPESLALFKRKDNLLEDYPFFGNKKRQDYPFFGNKKRQDYPFFGSRKRQNLREDKVDSSDDMWDFLERDIIPFWKRNRLASIKRSRMN

>TH2 endomorphin-like peptide prepropeptide

MDHKIKILALIVIAIAGLSSGKSMDKNGRNSVSLWTSAARDSKLAERNDQRKGYIYWETKRDENPESLALFKRKDNLLEDYPFFGNKKRQDYPFFGNKKRQDYPFFGSRKRQNLREDKVDSSDDMWDFLERDIIPFWKRNRLASIKRSRMN

>HH13 endomorphin-like peptide prepropeptide

MIHKIIIVALLVIAVTDLSAGKSMDGKKDEKTLSLWTSSLGSSKASRRNDQRNGYIYWETKRDNLPFFKRRNGYPFFGGKRESDYPFFGGKREVNIINFDYTYLYRAELWDMMKREGYPYWRRDRVAAYLRSKMI

>Ta-H1 FFNPamide prepropeptide

MKTLFILLVASVALPLIIAAKDESDSKAETNKRQFNPFFKKEAEVVITNSSVKLDASKAVKVARSEDNLQKKDDQFFNPGKRDDQFFNPDKRDDQFFNPGKRDDQFFNPGKRDDQFFNPGKRDDQFFNPGKRDDQFFNPGKRDGQFFNPGKRDGQFFNPGKRDGQFFNPGKRDGQFFNPGKRDDQFFNPGRRDDQFFHSRKYDGQFFNPGKREGQFFDKGKRDDQFFNPGKRDGQFFNPGKRDAQFFNPGRRYDTQFFSPDRRRDTQFFGQRSGKDEQFFGSRGDAQFFGSRRDGQFFNPGKRDAQFFGSRDDGQFFGSKKDDQFFGHKKEDDQFFGNKKDDAQFFRNNAEETPSYYSIPRAEFMHENSGTTNNDGNNCTCDGSAPVNPFFMY

>Ta-H2 FFNPamide prepropeptide

MKTLFILLVASVALPLIIAAKDESDSKAETNKRQFNPFFKKEAEDASKAVKVARSEDNLQKKDDQFFNPGKRDDQFFNPDKRDDQFFNPGKRDDQFFNPGKRDDQFFNPGKRDDQFFNPGKRDDQFFNPGKRDGQFFNPGKRDGQFFNPGKRDGQFFNPGKRDGQFFNPGKRDDQFFNPGRRDGQFFNPGKRDDQFFHSRKYDNQFFNPGKRDGQFFNPGKREGQFFDKGKRDDQFFNPGKRDGQFFNPGKRDAQFFNPGRRYDTQFFSPDRRRDTQFFGQRSGKADEQFFGSRGDAQFFGSRRDGQFFNPGKRDAQFFGSRDDGQFFGSKKDDQFFGHKKEDDQFFGNKKDDAQFFRNNAEETPSYYSIPRAEFMHENSGTTNNDGNNCTCDGSAPVNPFFVY

>Hoilungia-H13 FFGQamide prepropeptide

MKILFIFLIASMALPVIVSAKDESNEKSEINRRQFNPFFKKEVKVKIIIILFLIVSLLINLDDQFFGQGKRDAQFFGQGKRDDQFFGQGKRDDQFFGQGKRDDQFFGQGKRDDQFFGQGKRDDQFFGQGKREDQFFGQGKRDDQFFGQGKRDAQFFGQGKRDDQFFGQGKRNDQFFGQGKRDAQFFGQGKRDNQFFGQGKRDAQFFGQGKRDAQFFGQGKRDDQFFGQGKRDDQFFGQGKRDDQFFGQGKRDDQFFGQGKRDNQFFGQGKRDGQFFGNRHANKQFFGNGRGKFCLCFNMLHFMAYAVYV

>Cladtertia-H23 FFGQamide prepropeptide

MKILFIFLIASMALPAIISAKDEVNDKSEINRRQFNPFFKKEAKDTPKAAKAERSGMSTNFTINITSENLKQFSIILFNDHLMINFLAKANAMINFLDKANVVRIDFILVFIVKLRLKYSIFYSTDDQFFGQGKRDDQFFGQGKRDAQFFGQGKRDDQFFGQGKRDAQFFGQGKRDAQFFGQGKRDAQFFGQGKRDNQFFGQGKRDAQFFGQGKRDGQFFGNGRADKQFFGNGRDTQFFGNGRADTQFFGNGRDTQFFGNGRDTQFFGNRGDTQFFNPDRRDDAQFFGNRGDYQFFGNRDDGQFFGHKKDDQFFGSRKGI

PIIALLKQ

>Ta-H1 RWamide prepropeptide

MLTNRFIIWILFLGITTAQNVAKGKAQIGNHKSVFLKNEATRPERDQPPRWGRDQPTRWGRDQPPRWGRDQPPRWGRDQPSRWGRDQPPRWGRDQPPRWGRDQPPRWGRDQPPRWGRDQPPRWGRDQPPRWGRDQPPRWGRDQPPRWGRDQPPRWGRDQPPRWGRDQPPRWGRDQPPRWGGDQLPEMEKNHAPPRWGRDQYSWWNQEQYPSRWGREYSTPDNTAEKLLDSLTHQSENAKKNNFQEINSDSNSGNESAVHRLFSNKLKNQKAKSDSNKLMNSFSGSESISRPREKSLKRSETLDNMRIDLI

>Ta-H2 RWamide prepropeptide

MLTNRFIIWILFLGITTAQNVAKGKAQIGNHKSVFLKNEATRPERDQPPRWGRDQPTRWGRDQPPRWGRDQPPRWGRDQPSRWGRDQPPRWGRDQPPRWGRDQPPRWGRDQPPRWGRDQPPRWGRDQPPRWGRDQPPRWGRDQPPRWGRDQPPRWGGDQLPEIEKNYAPPRWGRDQYSWWNQEQYPSRWGREYSTPDNTAEKLLDSLTHQSENAKKNNFQEINSDSNSGNESAVHRLFSNKLKNQKAKSDSNKLMNSFSGSESISRPREKSLKRSETLDNMRIDLI

>Hoilungia-H13 RWamide prepropeptide

MLTNRLIILLLLGIATAKNVVKDNTAADVSDHSRFSKDQTYIIKSDQPPRWGRDQPPRWGRNQPPRWGRNQPLSWEYDQSLIYERDQPPRWGRDQPPRWGRNQPPRWGRDQPPRWGRNQPPRWGRDQPPRWGRDQPPRWGRDQPPRWGRDQPPRWGRDQPPRWGRDQPPRWGRDQPPRWGRNQPMELQVDHAPPRWGREQFSWWNEDKYPNRWGRKHHSSADNAKEESLDILMSQSKNTLDNMHKVIGTDNDAIVIGGLPSSINQANQDDKAATKTNDMTENLSVTE

>Cladtertia-H23 RWamide prepropeptide

MKMLANRLIILLLLGITTAQNVVKDKTVASIRDHSKLSKDTPYVVKRDQPPRWGRDQPPRWGRDQPPRWGRDQPPRWGRDQPPRWGRDQPPRWGRHQPPRWGRDQPPRWGRDQPPRWGRDQPPRWGRNQPPRWGRDDQPPRWGRDDQPPRWGRDDQPPRWGRDDQPPRWGRDDQPPRWGRDDQPPRWGRDDQPPRWGRDQPPRWGRSQPVELEDNQAPPRWGRDQFSWWNKDKYPNRWGRENHSSADKIRDESLDILTHQSKNMVDNIQKIIDGTNNDATIVDRLLSSKNQDDKATTKTNNVAEKLSLSE

>Ta-H1 WPPF prepropeptide

MYRLSLCCIIILVLFANEIEPKFAKPKEDIPWNLQRRSNANNLKSRSDSAKLSNTEHKKKDLVAEEQSHPIFGKGLVNEAKKTSRNEALQYNGWPPFRREDESKQYNGWPPFRREDESKQYNGWPPFRRSDELTQYNGWPPFRRNDGKEQYNGWPPFRRNAGMMQYNGWPPFRRDDEKMQYNGWPPFRREDREKQYNGWPPFRRDDEVMQYNGWPPFRRSEAVQYNGWPPFRRDDQQNKPYNGWPPFRREDQQNKPYNGWPPFRRDDQQNKPYNGWPPFRRNDQQKKPYNGWPPFRRNN

>Ta-H2 WPPF prepropeptide

MYRLSLCCIIILVLFANEIEPKFAKPKEDIPWNLQRRSNANNLKSRSDSAKLSNTEHKKKDLVAEEQSHPIFGKGLVNEAKKTSRNEALQYNGWPPFRREDESKQYNGWPPFRREDESKQYNGWPPFRRSDELTQYNGWPPFRRNDGKEQYNGWPPFRRNAGMMQYNGWPPFRRDDEKMQYNGWPPFRRDDDGKQYNGWPPFRRDDEVMQYNGWPPFRRSEAVQYNGWPPFRRDDQQNKPYNGWPPFRREDQQNKPYNGWPPFRRDDQQNKPYNGWPPFRRNDQQKKPYNGWPPFRRNN

>Hoilungia-H13 WPPF prepropeptide

MFRLPIYLTIVLVLCAHQIEPKHIKSKNDLLWNLQRRSHANLKAHSDLSKLANTEQKKKDSAAKEQTHPIFGKGDVKEAKASRDEISQYNGWPPFRRDNSKQYNGWPPFRSRSDAAAEVEQYNGWPPFRSRSDEMTEVEQYNGWPPFRRDDELKQYNGWPPFRREDQQNKPYNGWPPFRRDDQQNKPYNGWPPFRRDDQQNKPYNGWPPFRRDEQQNKPYNGWPPFRAIY
